# Current training provision and training needs in oral health for UK general practice trainees: survey of General Practitioner Training Programme Directors

**DOI:** 10.1186/s12909-016-0663-8

**Published:** 2016-05-11

**Authors:** Aneeta Ahluwalia, Tim Crossman, Helen Smith

**Affiliations:** Division of Primary Care and Public Health, Brighton and Sussex Medical School, Room 321 Mayfield House, Village Way, Brighton, BN1 9PH UK

**Keywords:** Oral health, Oral cancer, Survey, GP trainees, Education

## Abstract

**Background:**

In the UK the incidence of oral cancers has risen by a third in the last decade, and there have been minimal improvements in survival rates. Moreover, a significant proportion of the population no longer access dental health services regularly, instead presenting their oral health concerns to their General Medical Practitioner. Therefore, General Practitioners (GP) have an important role in the diagnosis of oral health pathologies and the earlier detection of oral cancers. This study aims to understand the current provision of training in oral health and cancer for GP trainees and to identify how unmet training needs could be met.

**Methods:**

A cross-sectional survey of GP Training Programme Directors using an online questionnaire asking about current oral health education training (hospital placements and structured teaching), the competencies covered with trainees and ways to improve oral health training. Quantitative data were analysed using descriptive statistics and content analysis was undertaken of free text responses.

**Results:**

We obtained responses from 132 GP Training Programme Directors (GPTPDs), from 13 of the 16 UK medical deaneries surveyed. The majority of respondents (71.2 %) indicated that their programmes did not provide any structured oral health training to GP trainees and that ≤ 10 % of their trainees were undertaking hospital posts relevant to oral health. GPTPDs were of the view that the quality of oral health training was poor, relative to the specified competencies, and that teaching on clinical presentations of ‘normal’ oral anatomy was particularly poor. It was envisaged that oral health training could be improved by access to specialist tutors, e-learning programmes and problem-based-learning sessions. Respondents highlighted the need for training sessions to be relevant to GPs. Barriers to improving training in oral health were time constraints, competing priorities and reluctance to taking on the workload of dentists.

**Conclusions:**

This UK-wide survey has identified important gaps in the training of GP trainees in relation to oral health care and cancer detection. Addressing these knowledge and skill gaps, particularly in the identification of oral cancers, will help to improve oral health and, more importantly, the timely diagnosis of oral cancer.

**Electronic supplementary material:**

The online version of this article (doi:10.1186/s12909-016-0663-8) contains supplementary material, which is available to authorized users.

## Background

Oral cancers include cancers of the lip, tongue, mouth, oropharynx, piriform sinus, hypopharynx, oral cavity and pharynx, of these it is cancers of the mouth and tongue that are the most common [1]. Despite some general improvements in oral health, oral cancer incidence rates in the UK have risen by a third in the last decade [1] (6,500 cases of oral cancer were diagnosed in 2011) and there have been minimal improvements in 5-year survival rates which remain around 50 % [2]. Its rising incidence and relatively low survival rates make oral cancer and oral health an important health issue. It was the 16^th^ most common cancer in the UK in 2011, accounting for more individual cancer diagnoses than liver, thyroid or cervical cancer [1]. Over 2,000 people died of oral cancer in the UK in 2012 [1]. Many oral cancers present at a late stage [3, 4] when the treatment required is more invasive and the survival rates lower. However research indicates that the majority of oral cancers are preceded by a detectable preclinical phase [5]. Despite the ease of access to the oral cavity for examination and early detection, most oral cancers are not diagnosed whilst localised [6].

A significant section of the UK population does not regularly access dental health services - in a 2015 survey 13 % of men, and 8 % of women reported not having been to a dentist in the previous five years [7]. Thus many patients with oral health concerns present initially to their general practitioner (GP). A survey of GP principals in Northeast England [8] found that nearly half were seeing between two and five patients each week with oral symptoms, and a similar number with dental or denture problems. General medical practitioners therefore have an important role to play in the early recognition and diagnosis of oral health problems [9, 10]. However, unlike dental practitioners, GPs express less confidence with conducting examinations of the oral cavity and diagnosing oral health pathologies [11–15].

The purpose of this study was to conduct a nationwide survey of General Practice Training Programme Directors (GPTPD) to understand the current training provision in oral health and oral cancer available for GP trainees. We wanted to ascertain the challenges to improving oral health training and better understand how unmet training needs could be addressed.

## Methods

The study was a cross-sectional internet-based survey of programme directors of general practice specialty training (GPST) programmes throughout the UK. The questionnaire (Additional file 1) was modelled on a previous survey of allergy training needs [16]. The questionnaire was anonymous, but respondents identified which deanery they worked in. The questions related to the teaching and training delivered between August 2012 and July 2013. GPTPDs were asked about the provision in their locality of oral health education for trainees through structured teaching and hospital placements. The survey described ten key competencies in oral health which were derived from the Royal College of General Practitioners (RCGP) curriculum relating to care of people with ENT oral and facial problems (section 3.15) at the time of undertaking this survey [17] (Table 1). Respondents were asked to rate on a Likert scale of one (non-existent) to ten (excellent) the training delivered by their programme in each key area. The GPTPDs were also asked if their trainees would benefit from additional training in oral health. They were asked to describe in free text the content, mode of delivery, and the support needed to achieve such expansion of oral health education for trainees. The survey was piloted prior to national distribution by GPST programme directors in our city.

**Table 1:** Learning outcomes and skills for care of people with ENT, oral and facial problems from the RCGP curriculum statement [17]

Primary care management	Know the epidemiology of head and neck cancers, including the risk factors, and identify unhealthy behaviour
	Understand how to recognise rarer but potentially serious conditions such as oral, head and neck cancer
Community orientation	Be aware of the need to refer patients with oral disease to appropriate specialist services in oral medicine or oral and maxillofacial surgery
A holistic approach	Understand that patients in poorer socio-economic situations (including the homeless) have higher rates of head and neck malignancy
	Know how community-specific aspects of oromucosal disease may be related to lifestyle (e.g. chewing paan, tobacco, betel nut, khat/qat, or reverse smoking)
Contextual features	Ensuring the practice welcomes patients from low socioeconomic classes and is active in reducing risk factors for head and neck malignancy
Attitudinal features	Avoiding a negative attitude towards homeless patients, which can lead to less vigilance in early detection of head and neck cancer in this group
Scientific features	Recognising that your training in ENT, oral and facial problems might need to be supplemented
	Demonstrating knowledge of the scientific backgrounds of symptoms, diagnosis and treatment of ENT, oral and facial conditions
	Understanding and implementing the key national guidelines that influence healthcare provision for ENT problems

Contact details of the General Practice Training Programme coordinators for the 16 Deaneries which provide GP training were obtained from the National Recruitment Office for General Practice Training website [18]. The 17^th^ deanery, the Defence deanery utilises other Deaneries for its GP training. We requested the programme coordinators cascade the questionnaire to all GPTPDs within their deanery. Responses were collected between November 2013 and February 2014. To optimise response rates, three reminder emails were sent to the GP Training Programme coordinators over this 4-month period.

### Data analysis

Quantitative data was analysed using descriptive statistics (Microsoft Excel 2010). The 95 % confidence intervals (CI) for proportions were calculated using an online calculator [19].

Respondents rated the quality of the oral cancer training on a Likert scale (ranging from 1 = extremely poor to 10 = excellent), the mean and standard deviation (SD) of responses for a given topic were calculated. Responses one to five were interpreted as signifying a ‘poor’ rating, consistent with previous research [16]. Content analysis of the free text data was undertaken by all authors [20]. Each author familiarised themselves with the data by reading and rereading the responses. They independently coded the free text responses, identifying key issues, concepts and themes before developing a common framework within which to analyse and interpret the data.

### Ethical considerations

In line with guidance from the National Health Service (NHS) Health Research Authority (HRA) on defining research [21], this survey is consistent with service evaluation; therefore submission to a Research Ethics Committee (REC) was not required. The programme directors were fully informed and participated voluntarily. All responses were confidential and anonymous.

## Results

Responses were obtained from 24.7 % (132/535) of all GPTPDs, providing feedback from 13 of the 16 UK deaneries surveyed.

### Clinical rotations to specialties relevant to oral health

Over a quarter (27.3 %; 36/132) reported that none of their GP trainees had the opportunity to undertake hospital posts relevant to oral health (for example ENT or Oral & Maxillofacial Surgery) in their training rotations. When GPTPDs reported some opportunity for trainees to undertake relevant hospital posts it was only available to the minority: 11–25 % of trainees in 14.4 % (19/132), less than 10 % of trainees in 58.3 % (77/132). Where there was no formal rotation some schemes had devised *ad hoc* arrangements, for example:

*‘We do not have an ENT post attached to our scheme. However, we do encourage our trainees to sit in the clinics when they can create some time’*

*‘Attend ENT clinics if they see a need’*

### Delivery of oral health training

The majority (71.2 %) of respondents (94/132) reported that their programme did not provide any oral health education for trainees through structured educational activities. Less than a fifth (18.2 %; 24/132) had formal structured oral health education training and 10.6 % were unsure. The teaching sessions described were mainly led by specialists (ENT or Oral & Maxillofacial Surgeons); others involved dentists, GPs with special interests (GPwSI) or dually qualified doctor/dentists. The format of teaching was predominantly lectures or seminars, however, two respondents described *‘trainee directed learning sessions’,* commenting *‘as their teaching is learner centred so it [*oral health*] may come up or may not. If it did it would be dealt with by problem based discussion, possibly topic teaching following some reading by one of the group’.*

### Quality of training

The majority of programme directors rated the quality of oral health training currently provided by their training programme with respect to each of the ten core oral health competencies as poor (Table 2). Concern was expressed about teaching of the clinical presentation of normal oral anatomy (90 % described this as non-existent or poor), the awareness of common dental problems (87 %) and the management and examination of the oral cavity (87 %). Nine out of ten respondents (89.4 %; 118/132) disagreed with the statement ‘I am confident that the oral health education currently being delivered by my programme meets all the curriculum requirements as listed’.

**Table 2:** General Practitioner Specialist Training programme directors’ opinions on the quality of training with respect to core oral health competencies measured on a 10-point Likert scale where 1 = non-existent and 10 = excellent teaching

Considering the oral health training currently provided by your programme, how would you arte the training delivered in each of these key areas:	Mean (SD)	Rated poor (scores 1–5) n/N	%	95 % CI
Examination of the oral cavity	3.3 (2.11)	113/130	87 %	81–93 %
Clinical presentations of ‘normal’ oral anatomy	2.8 (2.01)	117/130	90 %	85–95 %
Benign oral pathology	3.7 (2.33)	104/130	80 %	73–87 %
Oral manifestations of systemic disease	4.3 (2.23)	90/128	70 %	62–78 %
Awareness of common dental problems and their management	3.0 (2.08)	115/130	88 %	83–94 %
Oral cancer				
Epidemiology	4.0 (2.32)	93/127	73 %	66–81 %
- clinical presentation	4.5 (2.42)	86/128	67 %	59–75 %
- role of GP in diagnosis	4.4 (2.47)	89/128	70 %	62–78 %
- treatment modalities	3.2 (2.17)	108/127	85 %	79–91 %
Referral pathways of patients with oral disease	4.2 (2.52)	95/129	74 %	66–81 %

### Future oral health training for GP trainees

The vast majority of respondents (93.1 %; 123/132) agreed with the statement ‘our trainees would benefit from more training in oral health’. The preferred methods for enhancing oral health training were improved access to tutors with expertise in oral health and oral cancer (75 %), e-learning programmes (82 %) and example problem-based-learning (PBL) sessions (82 %). There was least support for expansion in the number of GPs with special interest in oral health (32.1 %) (Table 3). Some respondents felt prompted by the survey to address the unmet need and thanked us for drawing their attention to this topic:

**Table 3:** General Practitioner Specialist Training programme directors’ preferred approaches to enhancing oral health training for GP trainees

Preferred approaches to enhancing oral health training for GP trainees	n strongly agreeing or agreeing with approach described	%	95 % CI
Example problem-based learning sessions	107/130	82.3	76 %–89 %
Oral health e-learning programmes	106/130	81.5	75 %–88 %
Improved access to tutors with expertise in oral health and oral cancer	98/130	75.4	68 %–83 %
Support and teaching from outside bodies e.g. British Association of Oral and Maxillofacial Surgeons or British Dental Association	77/130	59.2	51 %–68 %
Funding	68/130	52.3	44 %–61 %
Increase in time for centralised teaching	51/130	39.2	31 %–47 %
Lecture notes	47/130	36.2	28 %–44 %
Expansion in number of GPs with special interest in oral health	42/131	32.1	24 %–40 %
Expansion in the number of oral health specialists	22/130	16.9	10 %–23 %

*‘Thanks for raising my awareness of this often marginalised area of the curriculum’*

*‘Great idea, I never considered that it doesn’t really feature in our training’*

However, other respondents were more guarded suggesting that the role of formal training could never be totally inclusive, emphasising the importance of teaching in the clinical setting and self-directed learning:

‘*The VTS* (vocational training schemes) *programmes cannot ever cover all aspects of all the curriculum statements and rely (correctly) on self-directed learning in the main’*

*‘Don’t forget that trainees learn a lot through their GP placement when they see patients and ask their trainers’*

*‘Oral health is important, but so are many other areas that receive little attention. The VTS shouldn't have to cover the whole curriculum and most of this area is covered in practice.’*

Programme directors wrote extensively about the practical barriers to incorporating teaching about oral health. They cited shortage of time, competing topics, prioritisation and opportunity costs, issues that were often interlinked:

*‘Ideally they would have more regular sessions in oral health, esp. oral cancer. But in practice these wd [sic] be competing with the myriad of other topics that specialists want GP training to cover from bed-wetting, diabetes, mental health etc., etc.***’**

*‘Obviously there is always scope for more training and oral conditions compete with numerous other curriculum areas. It might be helpful to ask TPDs to prioritise the field in relation to other topics’*

Not everyone considered oral health a priority area, making statements such as; *‘There are areas I would prioritise more than this’; ‘The course is so over packed it is difficult to see how this could be achieved without additional time or cutting back on more important areas’.* For some respondents the personal time pressures were so great they absolved all responsibility for enhancing oral health training saying, for example:

*‘Don't know, you tell us. Too busy to think let alone look at oral health***’**

Some respondents challenged the appropriateness of General Medical Practitioners developing additional skills in oral health and were concerned that they were being burdened to support a diminishing dental service:

‘*Oral cancer and oral manifestations of disease are important, but frankly it seems like this is a way of making up for the fact that dentistry is poorly run and expensive to access’*

*‘GPs have too much to do and their knowledge is spread too thinly across too many specialties. Dentists are best placed to deliver this type of care. Why should we learn even more stuff when other subjects are more central to our work and are not covered by other health professionals? I am constantly disappointed by the lack of support from dentists leaving us to manage their problems i.e. tooth abscess, antibiotic and analgesia prescribing, OOH (out of hours) dental pain’*

*‘We have enough of our own work to contend with, if there is not enough dental provision, that should be addressed, not try to dump their work on GPs’*

*‘I feel NHS dentists are trained to a much higher standard and should be utilised more’*

*‘Isn't oral health what Dentists are for? I would point to the GMC advice regarding managing dental issues when we are not qualified to do so. I am concerned that with the collapse of decent dental care in the UK, GPs are being (ab)used as the usual stopgap’*

Free text comments also highlighted the need for oral health training sessions to be *‘relevant’* and *‘targeted specifically for GPs’*, rather than aimed at hospital specialists. One respondent described a *‘successful session on ‘What a GP needs to know about dentistry’ given by an Oral & Maxillofacial surgeon last term-very well received by the trainees’.*

Their more familiar experience was of generic, untailored education, with less satisfactory results; ‘We *find ‘experts’ tend to take over teaching sessions and focus on their hospital specialist perspective. Improving teaching through GP specialists and support such as problem based learning* (PBL) *sessions and on-line resources would be most useful’.*

## Discussion

Our results indicate important gaps in the training of GP trainees in relation to oral health and oral cancer. The majority of trainees are not able to undertake hospital posts in ENT, Oral medicine or Oral & Maxillofacial Surgery during their training programme. Programme directors were of the view that the quality of oral health training was poor, relative to the specified competencies, and that teaching on clinical presentations of ‘normal’ oral anatomy was particularly poor. It was envisaged that oral health training could be improved by access to specialist tutors, e-learning programmes and problem-based-learning sessions. Respondents desired support from other specialists to help improve training but highlighted the need for training sessions to be relevant to GPs. Concerns were raised over time constraints and competing priorities within the curriculum. Some programme directors reflected on the need for trainees learning to be self-directed, emphasising the importance of the training that happened outside the taught programme, in the clinical setting. The programme directors’ general willingness to address knowledge gaps in oral health, particularly in the identification of oral cancers, should help to improve the future delivery of primary care-based oral health care.

GPTPDs expressed caution about taking on the workload of dentists; this was at times articulated as significant animosity between the discipline of medicine and dentistry. This is not necessarily a new finding; in 2006 when the new NHS dental contract was introduced GP leaders expressed concern that it would adversely affect GPs, with GPs being obliged to inappropriately pick up the work of dentists who had opted out of providing NHS care to their patients [22]. Seven years later these sentiments and concerns were still being expressed strongly by many of the respondents to our survey. Unsurprisingly, this inter-professional disrespect is bidirectional; a study published in 2010 that had interviewed Primary Care dentists about their experiences and views of the management of potentially malignant oral lesions by GMPs, found that dentists viewed GMPs’ management as poor, citing their frequent and inappropriate use of antibiotics [23]. Dentists also described their working relationship with GMPs as poor [23]. The interface between general dental practice and general medical practice needs attention to ensure patients with oral health problems do not suffer as their care and management fall through a professional and cultural gap.

### Strengths and limitations of this study

This is the first UK-wide survey of GPST oral health training provision. Its strengths are its robust design, adapting a survey design previously used in cross-sectional surveys of training needs [16]. Our survey was structured around the core competencies in oral health that are described in the RCGP curriculum. The assurance of confidentiality to respondents is apparent in the openness of responses and comments offered. However, our survey could be criticised for not soliciting responses from three (Northern, Northern Ireland and Oxford) of the 16 deaneries responsible for GP training in the UK. This may threaten generalisability but it is reassuring that the responding regions, although diverse in their demographics (221 to 1350 trainees (mean 781) and 9–61 GPTPD (mean 33)), were very similar and consistent in their responses. This exploratory study offers valuable insight into the issues around training in oral health, but we recognise that it is only from the perspective of programme directors. There is an urgent need for a parallel study that focuses on the GP trainees themselves, enabling us to better understand their oral health-related learning needs and styles as they undergo their specialist training.

### Implications for future research, policy and practice

This study highlights the need for additional expertise and teaching materials (including PBL sessions, online resources) to support GPST programmes in expanding their oral health education. Development of materials focussing on oral examination for red flags and criteria for specialist referral are a high priority. Once delivered, there is a need to establish whether such training is perceived as useful by those being trained and, importantly, whether this then translates into the much needed improvements in care provision for people with oral disease.

### Interpretation of findings in relation to previously published work

There is no comparable survey of the education provided to GPs in training but our study’s findings are consistent with studies of the confidence and readiness of qualified GPs to deal with oral health problems [12, 13]. A survey seeking to understand barriers to oral examination by UK general medical practitioners [15] found that two thirds (68 %) reported never having had training in ear, nose and throat conditions, and the majority (97 %) indicated that they had never had training in screening for oral cancer. The existing literature also highlights differences between GPs’ and dentists awareness of how to prevent and diagnose oral cancer, with GPs lacking confidence compared with that of their dental colleagues [13]. This disparity between the medical and dental professions is apparent in studies from other countries too; one American study demonstrating earlier stage at diagnosis of oral cancer when the referral originates from a dentist rather than a physician [24]. With respect to undergraduate teaching in the UK, studies have shown that just over 50 % of medical schools currently incorporate oral health within their curriculum [25, 26]. This arguably contributes to the lack of preparedness amongst qualified GPs, and threatens the feasibility of trainees acquiring their training from their trainers. Delegating the responsibility to GP trainers within day to day clinical practice may just perpetuate the problem.

## Conclusion

This UK-wide survey has identified important gaps in the training of GP trainees in relation to oral health care. Addressing these gaps, particularly in the identification of oral cancers, should help to improve delivery of primary care-based oral health care.

### Availability of data and materials

Data and materials are available on request from the corresponding author.

## Additional file

Additional file 1: PDF example of internet survey. Survey to identify the current training opportunities in oral health and oral cancer available for GP trainees. (DOCX 22 kb)

## Abbreviations

CI: confidence interval
ENT: ear nose & throat
GMC: General Medical Council
GMP: general medical practice
GP: general practitioner(s)
GPST: General Practice Specialty Training
GPTPD: General Practice Training Programme Director(s)
GPwSI: general practitioner with a special interest
NHS: National Health Service
RCGP: Royal College of General Practitioners
SD: standard deviation
