# Supplementary material for: Current training provision and training needs in oral health for UK general practice trainees: survey of General Practitioner Training Programme Directors
Source: BMC Med Educ. 2016 May 11;16:142. doi: 10.1186/s12909-016-0663-8 (PMC4863349; doi:10.1186/s12909-016-0663-8)

**Additional file 1 PDF example of internet survey**

**Survey to identify the current training opportunities in oral health and oral cancer available for GP trainees:**

Please answer the questions referring to the teaching delivered to your trainees from August 2012-July 2013. For the purposes of analysis all responses will be anonymous.

1. Which VTS region are you in?
2. Is your VTS training programme currently providing any oral health education for your trainees? Yes/No/Unsure
3. Who currently provides this training and what does it entail?


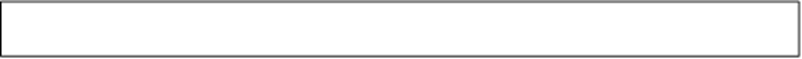


1. The Royal College of General Practitioners curriculum identifies key competencies in oral health which all trainees should achieve – These are listed below.

Considering the oral health training currently provided by your programme, how would you rate the training delivered in each of these key areas: (1 = extremely poor, 10= excellent)

1. Examination of the oral cavity

1 □ 2 □ 3 □ 4□ 5 □ 6 □ 7 □ 8 □ 9 □ 10 □

1. Clinical presentations of ‘normal’ oral anatomy (e.g. Fordyce spots, tori)

1 □ 2 □ 3 □ 4□ 5 □ 6 □ 7 □ 8 □ 9 □ 10 □

1. Oral cancer

Epidemiology: 1 □ 2 □ 3 □ 4□ 5 □ 6 □ 7 □ 8 □ 9 □ 10 □

Clinical presentation: 1 □ 2 □ 3 □ 4□ 5 □ 6 □ 7 □ 8 □ 9 □ 10 □

Role of GMP in diagnosis: 1 □ 2 □ 3 □ 4□ 5 □ 6 □ 7 □ 8 □ 9 □ 10 □

Treatment modalities: 1 □ 2 □ 3 □ 4□ 5 □ 6 □ 7 □ 8 □ 9 □ 10 □

1. Benign oral pathology (e.g. geographical tongue, recurrent apthous stomatitis)

1 □ 2 □ 3 □ 4□ 5 □ 6 □ 7 □ 8 □ 9 □ 10 □

1. Oral manifestations of systemic disease

1 □ 2 □ 3 □ 4□ 5 □ 6 □ 7 □ 8 □ 9 □ 10 □

1. Awareness of common dental problems and their management (dental abscess, acute pulpitis)

1 □ 2 □ 3 □ 4□ 5 □ 6 □ 7 □ 8 □ 9 □ 10 □

1. Referral pathways of patients with oral disease (e.g. when to refer to dentist or specialist)

1 □ 2 □ 3 □ 4□ 5 □ 6 □ 7 □ 8 □ 9 □ 10 □

1. I am confident that the oral health education currently being delivered by my programme meets all of the curriculum requirements – as listed above?

Strongly agree / agree/ neither agree nor disagree / disagree / strongly disagree

1. Our trainees would benefit from more training in oral health?

Strongly agree / agree/ neither agree nor disagree / disagree / strongly disagree

1. If you agree, please describe the training that you think is needed:


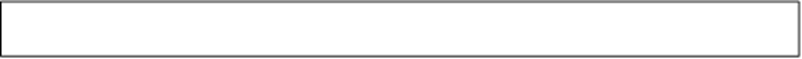


1. How could this best be delivered:


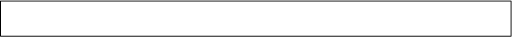


1. What support would your VTS training programme require to expand oral health education for your trainees?
2. Improved accessibility to tutors with expertise in oral health and oral cancer:

Expansion in the number of oral health specialists

(Strongly agree / agree/ neither agree nor disagree / disagree / strongly disagree)

1. Expansion in the no. of GPs with a special interest in oral health

(Strongly agree / agree/ neither agree nor disagree / disagree / strongly disagree)

1. Funding

(Strongly agree / agree/ neither agree nor disagree / disagree / strongly disagree)

1. Increase in time allocated for centralised teaching

(Strongly agree / agree/ neither agree nor disagree / disagree / strongly disagree)

1. Oral health e-learning programmes

(Strongly agree / agree/ neither agree nor disagree / disagree / strongly disagree)

1. Lectures notes

(Strongly agree / agree/ neither agree nor disagree / disagree / strongly disagree)

1. Example problem based learning sessions

(Strongly agree / agree/ neither agree nor disagree / disagree / strongly disagree)

1. Support and teaching from outside bodies e.g. British Association of Oral and Maxillofacial Surgeons (BAOMFS) or British Dental Association (BDA).

(Strongly agree / agree/ neither agree nor disagree / disagree / strongly disagree)

1. Do you have any additional comments?


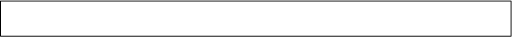

Supplement: Additional file 1: — PDF example of internet survey. Survey to identify the current training opportunities in oral health and oral cancer available for GP trainees. (DOCX 22 kb) [file 12909_2016_663_MOESM1_ESM.docx]
